# Fatty liver disrupts glycerol metabolism in gluconeogenic and lipogenic pathways in humans

**DOI:** 10.1194/jlr.M086405

**Published:** 2018-07-27

**Authors:** Eunsook S. Jin, Jeffrey D. Browning, Rebecca E. Murphy, Craig R. Malloy

**Affiliations:** Advanced Imaging Research Center,* University of Texas Southwestern Medical Center, Dallas, TX 75390; Department of Internal Medicine,† University of Texas Southwestern Medical Center, Dallas, TX 75390; Department of Clinical Nutrition,§ University of Texas Southwestern Medical Center, Dallas, TX 75390; Department of Radiology,** University of Texas Southwestern Medical Center, Dallas, TX 75390; Veterans Affairs North Texas Health Care System,†† Dallas, TX 75216

**Keywords:** tricarboxylic acid cycle, glucose, triglycerides, nonalcoholic fatty liver disease, nuclear magnetic resonance

## Abstract

It is a challenge to assess metabolic dysregulation in fatty liver of human patients prior to clinical manifestations. Here, we recruited obese, but otherwise healthy, subjects to examine biochemical processes in the liver with simple triglyceride accumulation using stable isotopes and NMR analysis of metabolic products in blood. Intrahepatic triglycerides were measured using ^1^H magnetic resonance spectroscopy, and volunteers received ^2^H_2_O and [U-^13^C_3_]glycerol orally, followed by a series of blood draws. NMR analysis of plasma triglycerides and glucose provided detailed information about metabolic pathways in patients with simple hepatic steatosis. Compared with subjects with low hepatic fat, patients with hepatic steatosis were characterized by the following: lower ^13^C enrichments in the glycerol backbones of triglycerides (i.e., TG-[^13^C]glycerol), higher [U-^13^C_3_]glycerol metabolism through the tricarboxylic acid (TCA) cycle, delayed gluconeogenesis from [U-^13^C_3_]glycerol, and less flexibility in adjusting supporting fluxes of glucose production upon an oral load of glycerol. In summary, simple hepatic steatosis was associated with enhanced [U-^13^C_3_]glycerol metabolism through pathways that intersect the TCA cycle and delayed gluconeogenesis from glycerol.

Nonalcoholic fatty liver disease (NAFLD) is the most common liver disease worldwide and affects more than 30% of the US population (1, 2). The disease is characterized by accumulation of intrahepatic triglycerides (IHTGs), and it is often associated with obesity and metabolic syndrome. Although generally asymptomatic, NAFLD encompasses a spectrum of disease activity ranging from simple steatosis to inflammation, fibrosis, and, potentially, cirrhosis. Simple hepatic steatosis is thought to be a benign condition characterized solely by IHTG accumulation in the absence of clinical indicators of liver disease (3). However, this bland condition may transition to nonalcoholic steatohepatitis in a subset of individuals, a more severe form of NAFLD that can lead to chronic liver disease (4). Early studies of hepatic metabolism in NAFLD depended in part on liver biopsies, but there is increasing emphasis on the use of stable isotope tracers. These approaches have demonstrated that lipid accrual pathways in liver, such as lipogenesis and lipolytic flux, are increased in NAFLD (5, 6). Simultaneously, the disposal of hepatic lipids via the secretion of triglycerides as VLDLs is increased in NAFLD; however, this process appears to be a saturable at IHTG values greater than ∼10% (7). Recently, oxidative lipid disposal has also been shown to be increased in affected individuals, occurring primarily via terminal oxidation of acetyl-CoA in the tricarboxylic acid (TCA) cycle (6, 8). This channeling of FAs toward the liberation of CO_2_ is associated with increased energy generation that directly supports the elevated rates of hepatic glucose production observed in NAFLD (6, 8). The use of stable isotopes has facilitated noninvasive studies of liver metabolism and enhanced our understanding of NAFLD; however, the aforementioned studies generally require prolonged continuous isotope infusions (∼2 h) that are not feasible in clinical environments or large patient populations.

Recently, we introduced a simple stable isotope method using ^13^C-labeled glycerol to study multiple biochemical processes in liver by analyzing plasma metabolic products. The method is undemanding for participants, requiring only an oral load of [U-^13^C_3_]glycerol followed by blood draws. Nonetheless, key metabolic processes are probed in the liver, including the pentose phosphate pathway (PPP), FA esterification, gluconeogenesis, and mitochondrial biosynthetic functions (9–11). These biochemical processes are all relevant in NAFLD and many other liver diseases; however, this method has not been applied in humans with liver disease. Here, we administered oral [U-^13^C_3_]glycerol to subjects with a range of hepatic fat, some with hepatic steatosis, measured by proton magnetic resonance spectroscopy (^1^H MRS). We recruited obese, otherwise healthy, volunteers who were divided into two groups according to IHTG content; low (≤ 5.0%) versus high (>5.0%) IHTG. All volunteers received both [U-^13^C_3_]glycerol and deuterium oxide (^2^H_2_O) to examine metabolic alterations in hepatic steatosis. We found that hepatic steatosis was associated with enhanced metabolism of glycerol through the TCA cycle prior to incorporation in triglycerides, delayed gluconeogenesis from [U-^13^C_3_]glycerol, and slower adjustment in glucose production after an oral load of glycerol. Hepatic metabolism of glycerol, an important substrate for both gluconeogenesis and lipogenesis, was significantly altered in human subjects with hepatic steatosis.

## MATERIALS AND METHODS

### Research design

This study was approved by the Institutional Review Board at the University of Texas Southwestern Medical Center. Each participant provided written informed consent prior to participation in accordance with the Declaration of Helsinki. Sixteen volunteers (ages, 22–59 years; BMI, 27–44 kg/m^2^; 2 males and 14 females; 11 Caucasians, 2 African Americans, and 3 mixed) completed this study. The characteristics of the study population are presented in **Table 1**. Ten female participants were premenopausal in the follicular phase of the menstrual cycle on the study day, and the remaining four were postmenopausal. Subjects with any chronic illness or use of medication aside from occasional antihistamines, aspirin, or nonsteroidal antiinflammatory drugs were excluded. All subjects had a meal (700 cal; 30% protein, 30% fat, and 40% carbohydrates) at 6:30 PM, started fasting at 7:00 PM, and received the first dose of ^2^H_2_O (70%) orally at 9:00 PM on the day prior to the study day. At 7:45 AM on the study day after the overnight fast, subjects were admitted to the Advanced Imaging Research Center located on the North Campus of the University of Texas Southwestern. After receiving the second dose of ^2^H_2_O, participants were directed to a 3T magnetic resonance (MR) scanner at 8:30 AM for ^1^H MRS to measure IHTG. After the scan, they received the third dose of ^2^H_2_O. The three doses of ^2^H_2_O were equally divided to minimize disequilibrium and vertigo in the association with rapid ingestion of ^2^H_2_O, and the total ^2^H_2_O dose was 5 g/kg body water (calculated as 60% of body weight for men or 50% of body weight for women) with a target of 0.5% in body water. An intravenous catheter was positioned in an antecubital vein, and blood (10 ml) was drawn for chemistry at baseline (*t* = −10 min). Subjects ingested [U-^13^C_3_]glycerol (50 mg/kg body weight; 50%; Sigma, St. Louis, MO) dissolved in water at 9:30 AM (t = 0), followed by a series of blood draws (30 ml each) at 30, 60, 90, 120, 180, and 240 min.

**TABLE 1.** Clinical and biochemical characteristics of subjects after an overnight fast

Characteristics	Low IHTG (n = 8)	High IHTG (n = 8)	*P*
IHTG (%)	1.8 ± 0.4	11.1 ± 2.0	<0.001
Male/female	1/7	1/7	
Age (year)	40 ± 4	46 ± 4	0.363
Body weight (kg)	88.0 ± 5.3	91.3 ± 5.1	0.659
BMI (kg/m^2^)	31.7 ± 1.8	34.4 ± 1.4	0.274
Hip circumference (cm)	109.4 ± 4.0	113.7 ± 5.2	0.485
Waist circumference (cm)	101.1 ± 3.7	106.5 ± 5.0	0.245
Blood test			
Partial thromboplastin time (s)	29.4 ± 0.8	27.1 ± 0.6	0.042
INR	1.00 ± 0.00	0.98 ± 0.02	0.149
HgbA1c (%)	5.5 ± 0.1	5.6 ± 0.1	0.609
Total cholesterol (mmol/l)	4.7 ± 0.4	5.3 ± 0.2	0.248
HDL-cholesterol (mmol/l)	1.3 ± 0.1	1.4 ± 0.1	0.556
LDL-cholesterol (mmol/l)	3.0 ± 0.3	3.3 ± 0.2	0.449
AST (U/l)	17 ± 2	17 ± 2	0.791
ALT (U/l)	19 ± 7	20 ± 3	0.974
GGT (U/l)	22 ± 6	19 ± 2	0.603
Insulin (μU/ml)	17.3 ± 7.3	9.14 ± 3.8	0.607

Values are mean ± SE. ALT, alanine aminotransferase; AST, aspartate aminotransferase; GGT, γ-glutamyl transferase; INR, international normalized ratio.

### IHTG measurement and NMR for plasma triglyceride and glucose analysis

^1^H MRS of liver was performed using a 3 T Achieva whole-body MR system (Philips Medical Systems) as described previously (8). Plasma lipids were extracted and plasma glucose was derivatized to monoacetone glucose (MAG) for NMR acquisition as described previously (9). All NMR spectra were collected using a Varian INOVA 14.1 T spectrometer (Agilent, Santa Clara, CA) equipped with a 3 mm broadband probe with the observe coil tuned to ^13^C (150 MHz). ^13^C NMR spectra of lipids were collected using a 60° pulse, a sweep width of 36,765 Hz, 110,294 data points, and a 1.5 s acquisition time with 1.5 s interpulse delay at 25°C. Proton decoupling was performed using a standard WALTZ-16 pulse sequence. Spectra were averaged for ∼23,000 scans requiring 20 h. The solvent (CDCl_3_) resonance was set to 77.2 ppm. ^13^C and ^2^H NMR spectra of MAG were collected as described previously (12). All NMR spectra were analyzed using the ACD/Labs NMR spectral analysis program (Advanced Chemistry Development, Inc., Toronto, Canada).

### NMR analysis of the glycerol backbones of triglycerides and glucose

**Fig. 1.**
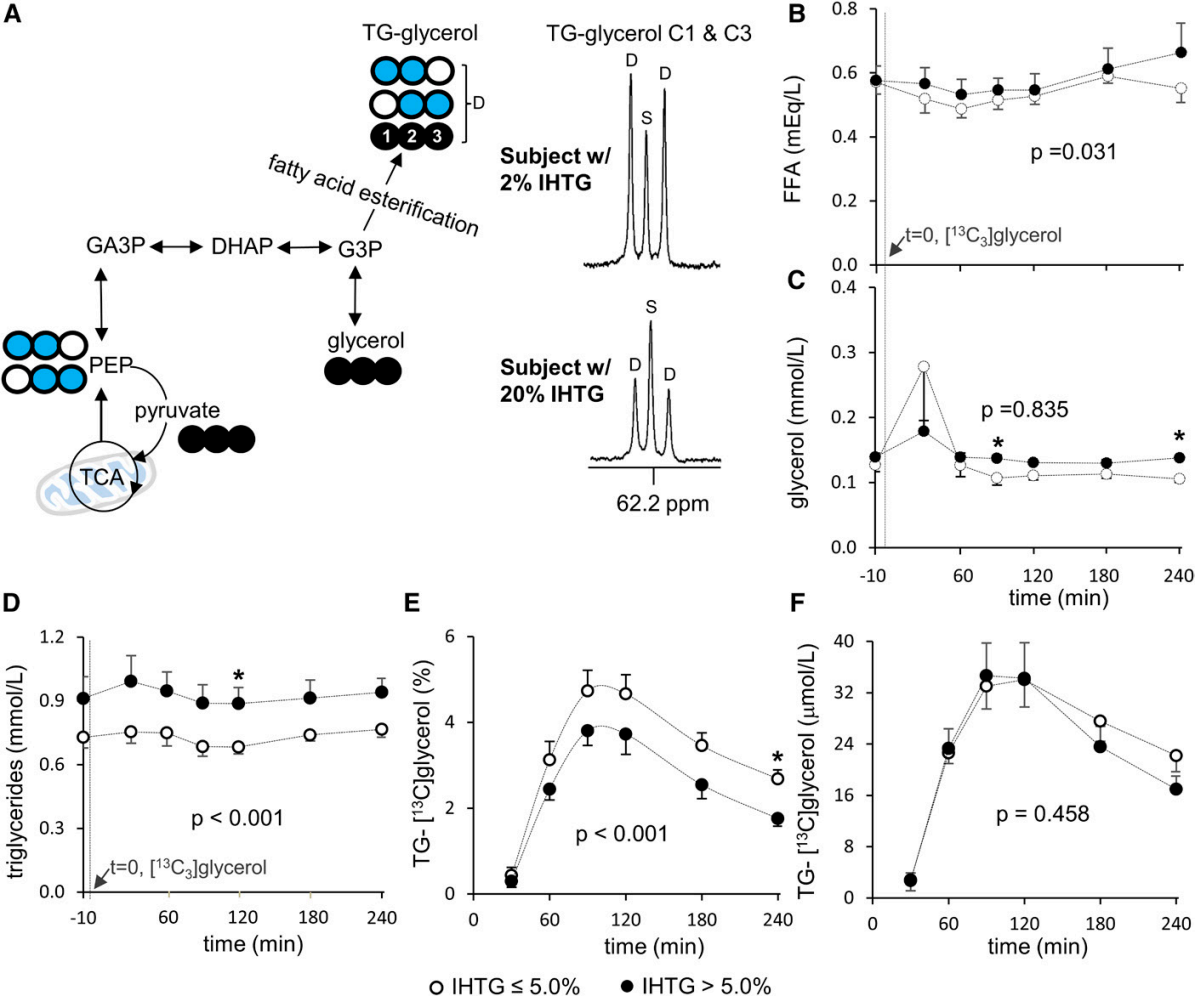
Plasma TG-glycerol analysis after an oral load of [U-^13^C_3_]glycerol. A: In liver, phosphorylated [U-^13^C_3_]glycerol is utilized for FA esterification or metabolized through the TCA cycle prior to glyceroneogenesis. Because triglycerides are released into the circulation, ^13^C-labeling patterns in TG-glycerol can be analyzed using plasma lipid extracts. In TG-glycerol C1 and C3 resonance of ^13^C NMR, a doublet (D) reflects excess ^13^C-lebeled glycerol backbones of triglycerides. B: Plasma FFA concentration is slightly higher in the high compared with the low-IHTG group. C: Plasma glycerol concentration is similar between the groups. D: Plasma triglyceride concentration is higher in the high compared with the low-IHTG group. E, F: The enrichment of TG-[^13^C]glycerol (%) is lower in the high than the low-IHTG group, but the absolute concentration of TG-[^13^C]glycerol is the same between the groups. Open circle, ^12^C; black circle, ^13^C; blue circle, ^13^C after metabolism through the TCA cycle; n = 6–8 at each time point. * *P* < 0.05 at each time point. *P* value in each graph was determined by two factor ANOVA with replication analysis. DHAP, dihydroxyacetone phosphate; GA3P, glyceraldehyde 3-phosphate; PEP, phospho*enol*pyruvate.

In ^13^C NMR of plasma lipid extracts (Fig. 1), the doublet (D) signal from the glycerol backbones of triglycerides (TG-glycerol) carbons 1 and 3 (C1 and C3) at 62.2 ppm reflects both double-labeled (●●○ and ○●● [1,2-^13^C_2_] and [2,3-^13^C_2_], respectively) and triple-labeled (●●● [U-^13^C_3_]) glycerol backbones, whereas the singlet (S) reflects single-labeled glycerol backbones with natural abundant ^13^C (1.1%; ●○○, ○○● ([1-^13^C_1_] and [3-^13^C_1_], respectively). The ^13^C enrichment in TG-glycerol (percent) was calculated by assuming the area under the singlet as natural abundant ^13^C due to the low probability of forming a single-labeled glycerol from [U-^13^C_3_]glycerol. The absolute concentration of triglycerides containing ^13^C-labeled glycerol backbones (TG-[^13^C]glycerol) in plasma was measured by multiplying the fraction of ^13^C enrichment in TG-glycerol with triglyceride concentration.

**Fig. 2.**
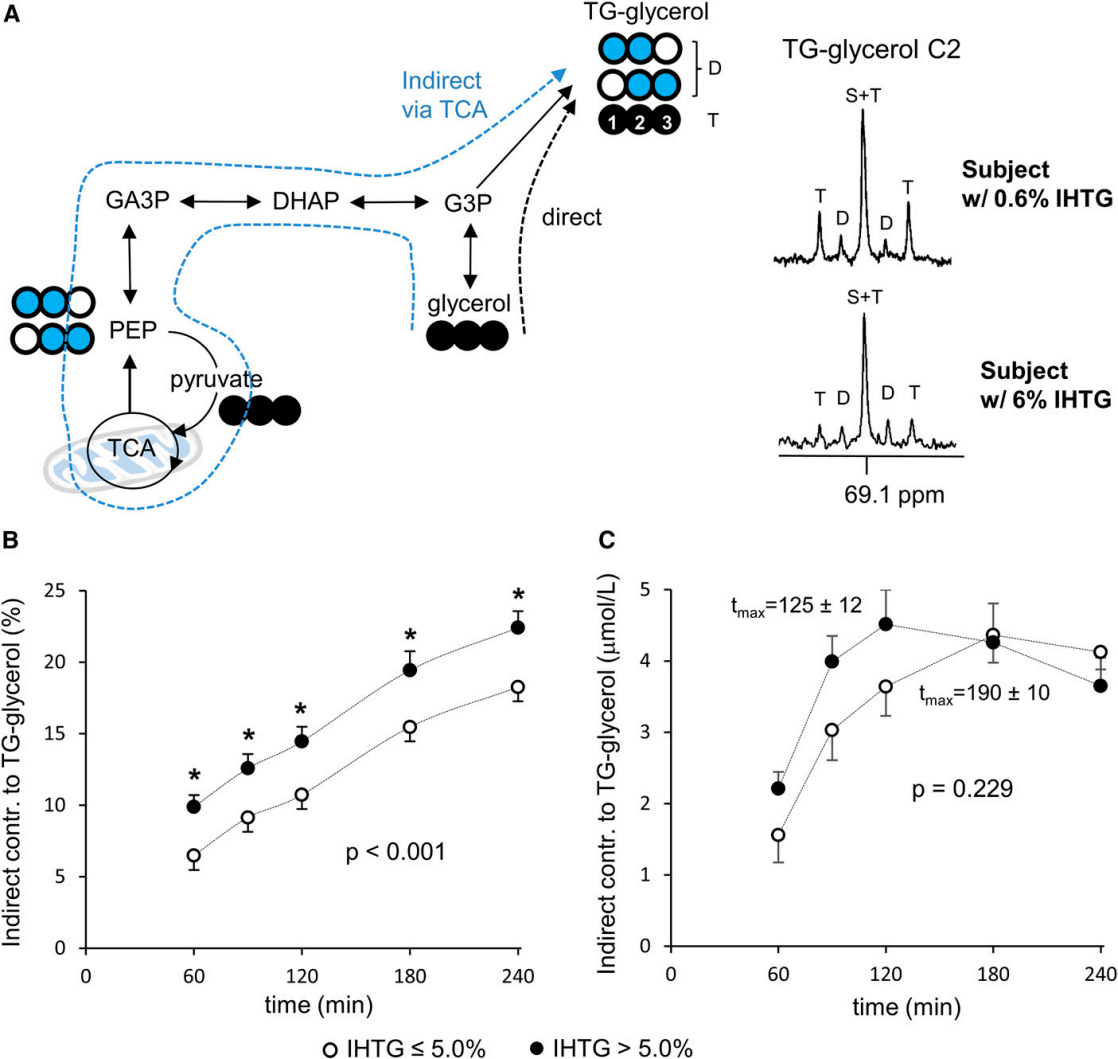
Enhanced [U-^13^C_3_]glycerol metabolism through the TCA cycle in fatty liver. A: In TG-glycerol C2 resonance of ^13^C NMR, a doublet (D) reflects [U-^13^C_3_]glycerol metabolism through the TCA cycle prior to triglycerides (i.e., indirect contribution) while a triplet (T) reflects direct [U-^13^C_3_]glycerol incorporation into triglycerides. B: The fraction of indirect contribution is higher in the high compared with the low-IHTG group. C: The absolute concentration of TG-[^13^C_2_]glycerol (i.e., triglycerides containing ^13^C-glycerol backbones incorporated through the TCA cycle) is similar between the groups (*P* = 0.229). However, the time to peak concentration (*t*_max_) is faster in the fatty liver group by 65 min (*P* = 0.002; 190 ± 10 min in the low vs. 125 ± 12 min in the high-IHTG group). Open circle, ^12^C; black circle, ^13^C; blue circle, ^13^C after experiencing the TCA cycle; n = 6–8 at each time point. * *P* < 0.05 at each time point. *P* value was determined by two factor ANOVA with replication analysis.

In ^13^C NMR of TG-glycerol C2 at 69.1 ppm (Fig. 2), five resonance components are made of a singlet (S), a doublet (D), and a triplet (T). Unlike TG-glycerol C1 and C3 resonance, triple-labeled and double-labeled glycerol backbones are distinguished by the T (1:2:1) and the D (1:1) in C2 resonance. Here, the S and the middle peak of the T are overlapped (S+T), but the portion of each component can be calculated because the middle peak area of the T equals the sum of two lateral peak areas. The T and D provide information about the incorporation of [U-^13^C_3_]glycerol into triglycerides via the direct [glycerol → glycerol 3-phosphate (G3P) → TG-glycerol] and indirect [glycerol → G3P → triose pool → phospho*enol*pyruvate (PEP) → pyruvate → TCA cycle → PEP → triose pool → G3P → TG-glycerol] pathways, respectively. The indirect contribution of [U-^13^C_3_]glycerol to TG-glycerol was calculated by D/(D+T), whereas the direct contribution was by T/(D+T). The indirect contribution reported in the present work is a low detection limit because triple-labeled glycerol backbones of triglycerides (TG-[^13^C_3_]glycerol) produced through the TCA cycle is reported as the direct contribution (9). Consequently, the direct contribution based on the appearance of TG-[^13^C_3_]glycerol could be overestimated.

In ^13^C NMR analysis of MAG derived from plasma glucose, two S resonances from methyl groups are internal references to measure ^13^C enrichments in glucose (13). The methyl groups are added during glucose conversion to MAG, and the carbons of the groups are natural abundant ^13^C. As an example, the enrichment by [5,6-^13^C_2_]glucose was calculated using the ratio of D signal of the glucose (D56) at 70.8 ppm over the sum of two S areas from methyl resonances (2S_methyl_; 26.1 and 26.7 ppm). Any increase in the ratio of D56/2S_methyl_ over the natural abundance reflects excess ^13^C enrichment by [5,6-^13^C_2_]glucose. The overall ^13^C enrichment in glucose is the sum of individual enrichment by each glucose isotopomer with excess ^13^C. The concentration of ^13^C-labeled glucose in plasma is calculated by multiplying the fraction of enrichment with plasma glucose concentration.

### Plasma metabolite and hormone assay

Cholesterol, hemoglobin A1c (HgbA1c), transaminases, and other routine blood tests were performed by a commercial laboratory (Quest Diagnostics). FFAs and triglycerides were measured using the Vitros 250 analyzers (Johnson and Johnson), glucose was measured using glucose oxidase (YSI 2300 Glucose Analyzer; GMI, Inc), insulin was measured using an ELISA kit (Millipore Co.), and free glycerol was measured using a commercial kit (Sigma).

### Statistical analysis

Data are expressed as mean ± standard error. Comparisons between the low- and high-IHTG groups with repeated measurements over time were made using two-way ANOVA with replication. Comparisons between two sets of data were made using one-way ANOVA, where *P* < 0.05 was considered significant.

## RESULTS

### [U-^13^C_3_]glycerol incorporation to triglycerides through the TCA cycle is enhanced in fatty liver

All volunteers received [U-^13^C_3_]glycerol and ^2^H_2_O orally followed by a series of blood draws for NMR analysis of metabolic products. After phosphorylation by glycerol kinase in liver, [U-^13^C_3_]glycerol can be used for FA esterification, resulting in TG-[^13^C]glycerol. The direct incorporation of [U-^13^C_3_]glycerol produces TG-[^13^C_3_]glycerol, whereas [U-^13^C_3_]glycerol metabolism through the TCA cycle prior to triglycerides produces double-labeled glycerol backbones (TG-[^13^C_2_]glycerol). This information is encoded in newly esterified triglycerides secreted by the liver and available in circulating plasma. **Fig. 1A** shows ^13^C NMR of TG-glycerol C1 and C3, which was analyzed for ^13^C enrichments in the glycerol backbones. The concentrations of plasma FFAs and glycerol during the study period were similar between the low- and high-IHTG groups, but the concentration of triglycerides was higher in the high-IHTG group compared with the low group (Fig. 1B–D). The enrichment of TG-[^13^C]glycerol (percent) in plasma was lower in the high-IHTG group after ingestion of [U-^13^C_3_]glycerol (Fig. 1E); however, the absolute plasma concentration of TG-[^13^C]glycerol did not differ between the groups (Fig. 1F).

In the TG-glycerol C2 region (**Fig. 2A**), it is easy to appreciate that the D/T ratio was higher in the high-IHTG group. Indeed, calculated fractional indirect contribution of [U-^13^C_3_]glycerol to triglycerides was higher by ∼3–4% in the high-IHTG group at each time point evaluated (Fig. 2B). The time to peak concentration (*t*_max_) of TG-[^13^C_2_]glycerol was also faster in the high-IHTG group by 65 ± 12 min (*P* = 0.002; Fig. 2C). These data indicate increased and more rapid shunting of glycerol toward the TCA cycle in those with fatty liver.

### Gluconeogenesis from [U-^13^C_3_]glycerol is delayed in subjects with fatty liver

**Fig. 3.**
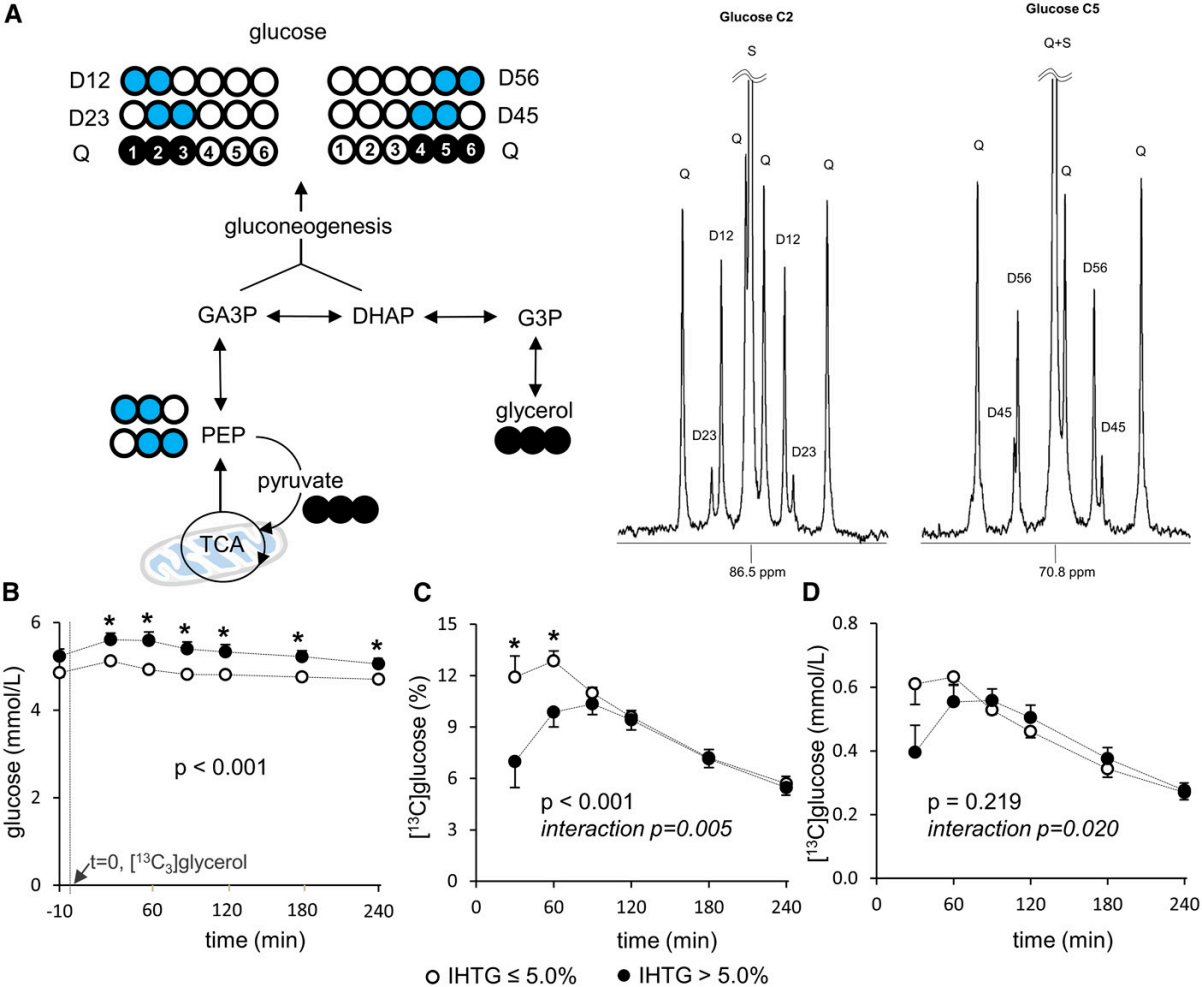
Delayed [U-^13^C_3_]glycerol incorporation to gluconeogenesis in fatty liver. A: [U-^13^C_3_]glycerol direct incorporation to gluconeogenesis produces triple-labeled ([1,2,3-^13^C_3_] or [4,5,6-^13^C_3_]) glucose, whereas indirect contribution through the TCA cycle produces double-labeled ([1,2-^13^C_2_], [2.3-^13^C_2_], [4.5-^13^C_2_], or [5,6-^13^C_2_]) glucose. All signals from these glucose isotopomers are observed in ^13^C NMR of a glucose derivative. B: Plasma glucose is slightly higher in the high compared with the low-IHTG group after [U-^13^C_3_]glycerol administration. C: The ^13^C-enrichment in plasma glucose (percent) is lower up to 60 min, and peak enrichment is also lower in the high compared with the low-IHTG group. D: A similar, but less striking, pattern is observed in the absolute concentration of [^13^C]glucose. Open circle, ^12^C; black circle, ^13^C; blue circle, ^13^C after experiencing the TCA cycle; n = 8 at each time point. * *P* < 0.05 at each time point. *P* value and interaction *P* value were determined by two-factor ANOVA with replication analysis.

After the administration of [U-^13^C_3_]glycerol, ^13^C labeling patterns of plasma glucose provide information about multiple metabolic processes in liver. Gluconeogenesis directly from [U-^13^C_3_]glycerol produces triple-labeled ([1,2,3-^13^C_3_] and [4,5,6-^13^C_3_]) glucose, which was dominant in all subjects (**Fig. 3A**). Similarly, as noted in triglycerides, double-labeled glucose isotopomers reflect [U-^13^C_3_]glycerol metabolism through the TCA cycle in mitochondria. Plasma glucose was modestly, but significantly, higher in the high compared with the low-IHTG group after the glycerol load (Fig. 3B). The ^13^C enrichment of glucose (percent) was lower in the high-IHTG group by up to 60 min, and peak enrichment was also lower compared with the low-IHTG group (Fig. 3C). A similar, but less striking, pattern was observed for the absolute concentration of ^13^C-enriched glucose over time (Fig. 3D). Given that values between the groups for both measures were similar beyond the 60 min time point, this finding did not appear to be related to any differences in glucose pool size.

**Fig. 4.**
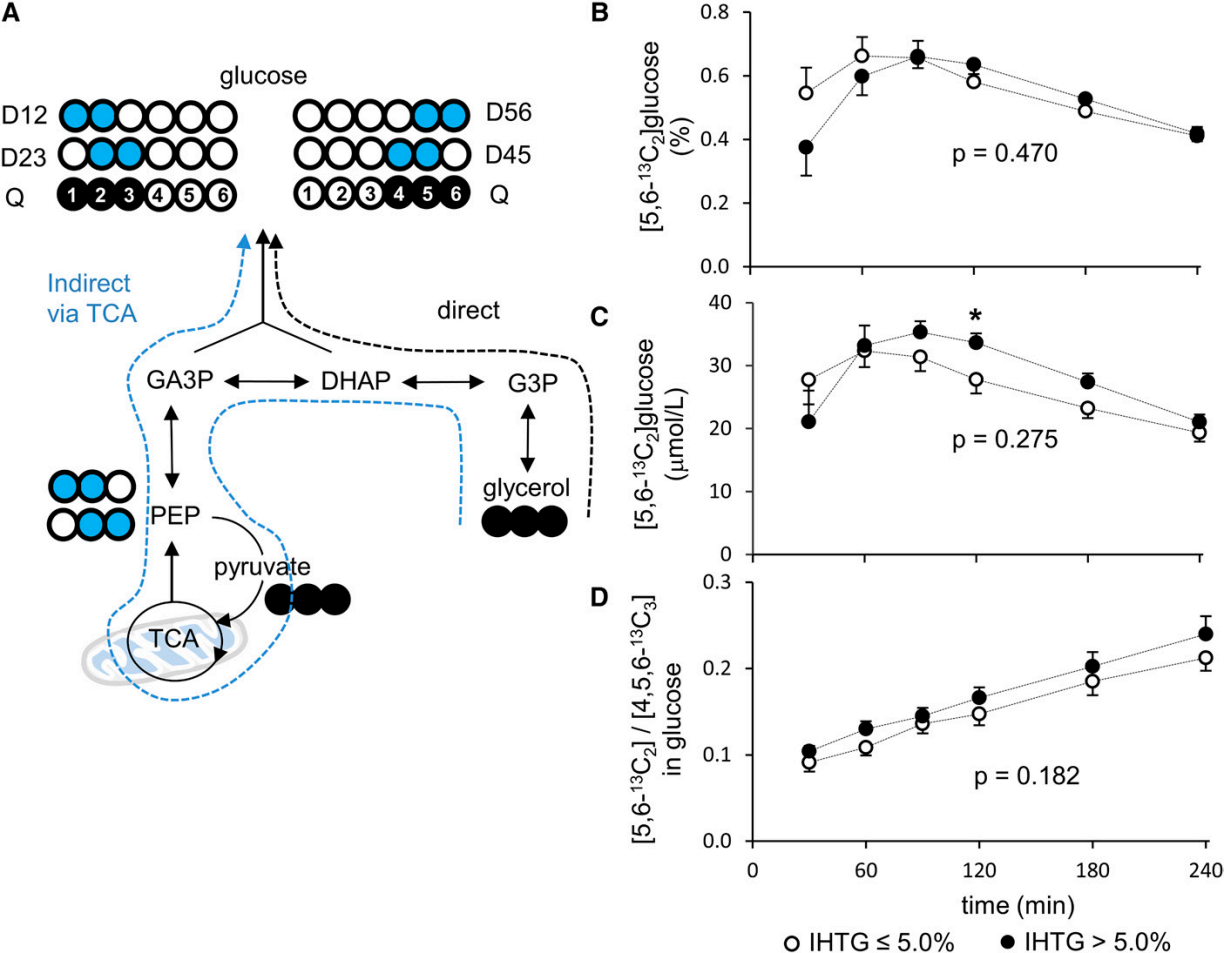
[U-^13^C_3_]glycerol metabolism through the TCA cycle prior to gluconeogenesis. A: [5,6-^13^C_2_]glucose is evidence of [U-^13^C_3_]glycerol metabolism through the TCA cycle prior to gluconeogenesis. B: The fraction of [5,6-^13^C_2_]glucose (percent) in plasma is similar between the low and high-IHTG groups. C: Overall the absolute concentration of [5,6-^13^C_2_]glucose (μmol/l) is not altered in the high-IHTG group. However, the concentration at the 120 min time point is higher in the high compared with the low-IHTG group. D: The ratio of [5,6-^13^C_2_]/[4,5,6-^13^C_3_] in glucose reflects the fraction of [U-^13^C_3_]glycerol that passes through the TCA cycle prior to gluconeogenesis. The ratio tends to be higher in the high compared with the low-IHTG group without statistical significance. Open circle, ^12^C; black circle, ^13^C; blue circle, ^13^C after experiencing the TCA cycle; n = 8 at each time point. * *P* < 0.05 at each time point; *P* value was determined by two-factor ANOVA with replication analysis.

Among double-labeled ([1,2-^13^C_2_], [2,3-^13^C_2_], [4,5-^13^C_2_], and [5,6-^13^C_2_]) glucose isotopomers, [5,6-^13^C_2_]glucose is a useful marker for [U-^13^C_3_]glycerol metabolism through the TCA cycle (**Fig. 4A**). The ^13^C-labeling patterns in glucose carbons 4–6 are independent from hepatic PPP activity, and the signal from [5,6-^13^C_2_]glucose is greater than that from [4,5-^13^C_2_]glucose. Overall both the enrichment (percent) and the concentration (μmol/l) of [5,6-^13^C_2_]glucose were not different between the two groups (Fig. 4B, C). However, the concentration at the 120 min time point was higher in the high compared with the low-IHTG group. The ratio of [5,6-^13^C_2_]/[4,5,6-^13^C_3_] in glucose, a reflection of the fraction of [U-^13^C_3_]glycerol that passed through the TCA cycle prior to utilization for gluconeogenesis, tended to be higher in the high compared with low-IHTG group without statistical significance at all postload time points (Fig. 4D).

**Fig. 5.**
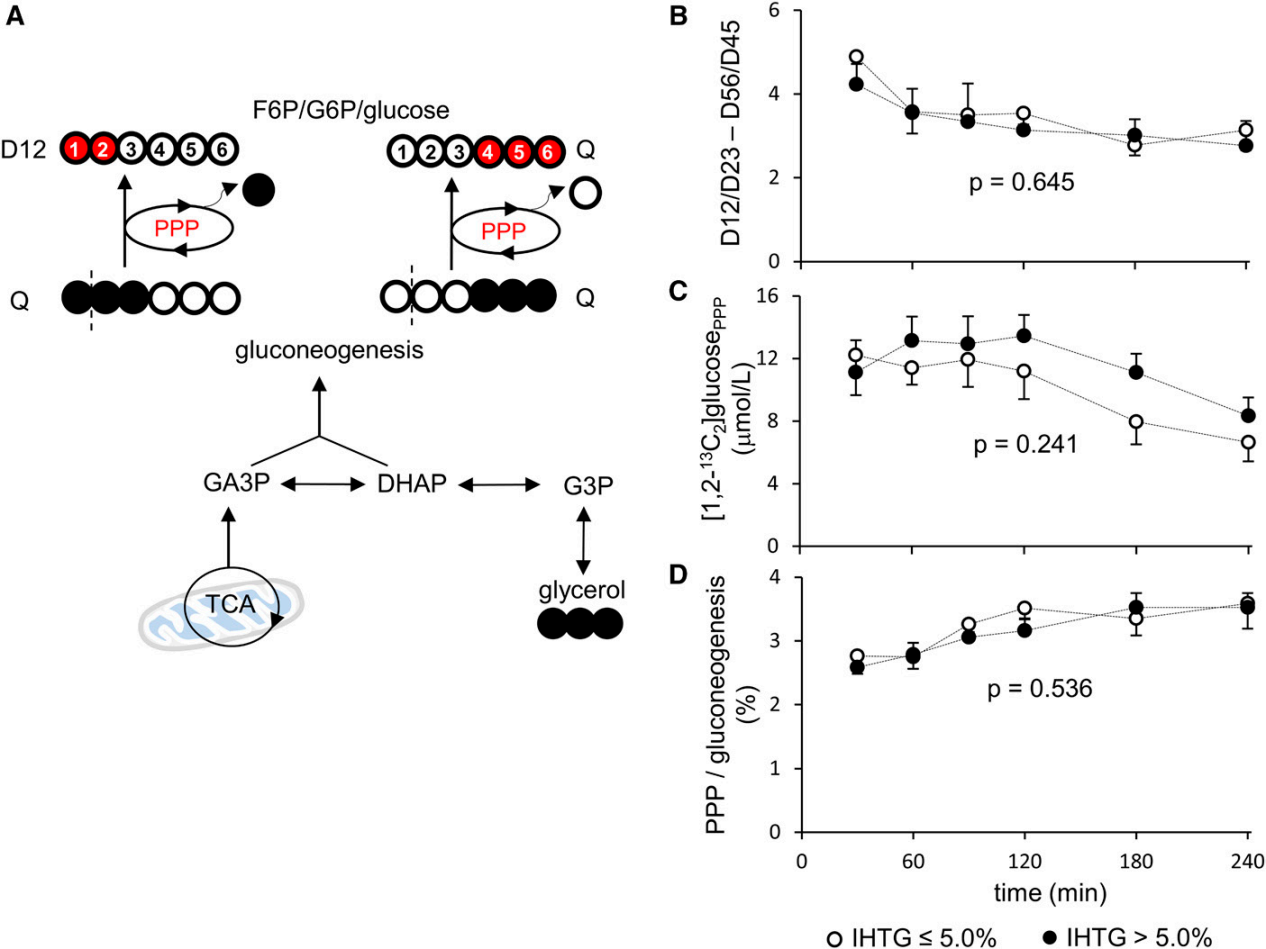
Hepatic PPP activity. A: Gluconeogenesis directly from [U-^13^C_3_]glycerol produces [1,2,3-^13^C_3_] or [4,5,6-^13^C_3_]hexose. When these hexose isotopomers enter the full cycle of PPP, [1,2-^13^C_2_] and [4,5,6-^13^C_3_]hexose are produced from [1,2,3-^13^C_3_] and [4,5,6-^13^C_3_]hexose, respectively. Note that the labeling pattern of [4,5,6-^13^C_3_]hexose remains the same, whereas double-labeled, [1,2-^13^C_2_]hexose is produced from [1,2,3-^13^C_3_]hexose through the PPP activity. Thus, the PPP activity leads to ratio difference between [1,2-^13^C_2_]/[2,3-^13^C_2_] and [5,6-^13^C_2_]/[4,5-^13^C_2_] in glucose. B, C: The ratio difference and the concentration of [1,2-^13^C_2_]glucose produced through the PPP are similar between the low and high-IHTG groups. D: PPP flux relative to gluconeogenesis is also similar between the groups. Open circle, ^12^C; black circle, ^13^C; red circle, ^13^C after experiencing the PPP; n = 7 or 8 at each time point; *P* value was determined by two-factor ANOVA with replication analysis.

Assessment of hepatic PPP activity was based on [1,2-^13^C_2_]glucose production through the PPP (10). As noted, [1,2,3-^13^C_3_] and [4,5,6-^13^C_3_]hexose are produced through gluconeogenesis directly from [U-^13^C_3_]glycerol. When [1,2,3-^13^C_3_] and [4,5,6-^13^C_3_]hexose enter the PPP, [1,2-^13^C_2_] and [4,5,6-^13^C_3_]hexose are produced as a result of decarboxylation of C1 in the oxidative phase of this pathway, followed by pentose carbon rearrangement in the nonoxidative phase (**Fig. 5A**). This leads to a difference in the ratios of [1,2-^13^C_2_]/[2,3-^13^C_2_] and [5,6-^13^C_2_]/[4,5-^13^C_2_] in glucose. Using the ratio difference, the PPP activity can be reflected by the amount of [1,2-^13^C_2_]glucose produced through the PPP ([1,2-^13^C_2_]glucose_PPP_) and PPP flux relative to gluconeogenesis (PPP/gluconeogenesis). However, none of these measurements was different between the two groups (Fig. 5B–D). These data indicate that the PPP activity was not altered in individuals with subclinical NAFLD.

### Fatty liver is slower in adjusting supporting pathways of glucose production after an oral load of glycerol

**Fig. 6.**
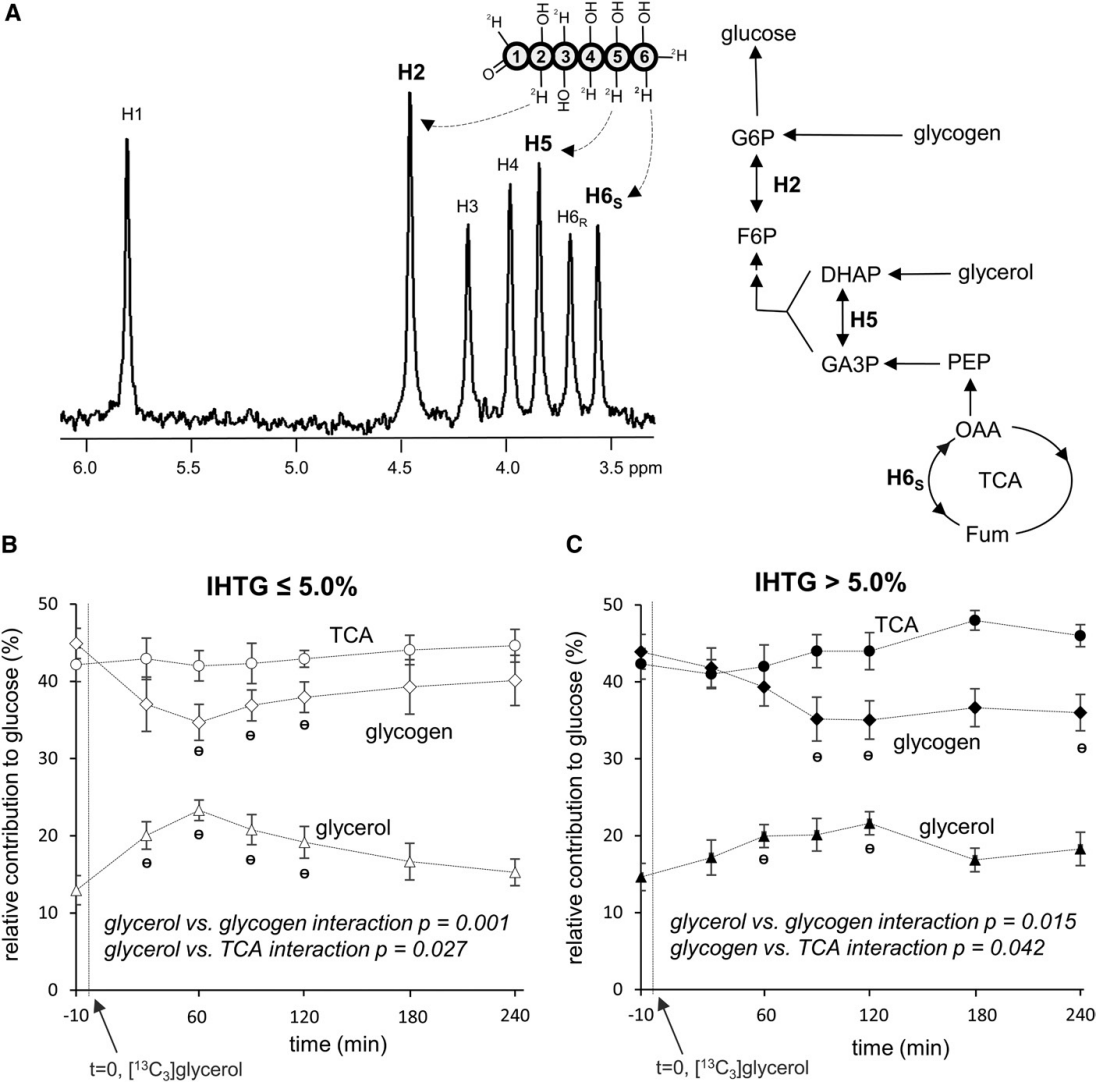
Sources of glucose after [U-^13^C_3_]glycerol administration. A: In the presence of ^2^H_2_O, glucose may be labeled at H2 [glucose 6-phosphate (G6P) ↔ fructose 6-phosphate (F6P)], H5 [dihydroxyacetone phosphate (DHAP) ↔ glyceraldehyde 3-phosphate (GA3P)], and H6s (oxaloacetate ↔ fumarate) positions depending on its source, informing relative contributions from glycogen, glycerol, and the TCA cycle. Glucose derived from the TCA cycle is reflected by ^2^H (deuterium) signal at the H6s position, glucose derived from glycogen by the signal difference in H2 and H5 positions, and glucose derived from glycerol by the signal difference in H5 and H6s positions. B: In the low-IHTG group, an oral load of [U-^13^C_3_]glycerol temporarily increases relative glycerol contribution, which is accompanied by a decrease in the contribution of glycogen. The contribution from the TCA cycle remains unchanged. C: In the high-IHTG group, the load also increases the glycerol contribution accompanying glycogen contribution decrease, but the response is slower compared with the low-IHTG group. n = 7 or 8 at each time point. ^Ɵ^ *P* < 0.05 compared with the value at *t* = −10 min; interaction *P* value was determined by two-factor ANOVA interaction analysis.

The distribution of deuterium (^2^H) in plasma glucose after ^2^H_2_O administration is sensitive to relative contributions from glycogen, glycerol, and precursors originating from the TCA cycle to glucose production (14, 15). In the ^2^H NMR spectrum of a glucose derivative shown in **Fig. 6A**, the TCA cycle contribution is reflected by the ^2^H signal of glucose H6_s_ position, glycogen contribution by the difference in ^2^H signal between H2 and H5, and glycerol contribution by the difference in ^2^H signal between H5 and H6_s_. In comparing relative contributions at each time point, both groups had similar contributions from each source; 41–48% from the TCA cycle, 35–45% from glycogen, and 13–23% from glycerol (Fig. 6B, C). An oral [U-^13^C_3_]glycerol load temporarily increased glycerol contribution in both groups (13% → 23% at 60 min in the low-IHTG group and 15% → 22% at 120 min in the high-IHTG group), which was accompanied by a decrease in the contribution of glycogen. Interestingly, the high-IHTG group was slower in responding to the glycerol load compared with the low-IHTG group. Whereas the low-IHTG group had a maximum glycerol contribution at 60 min, the high-IHTG group reached a maximum at 120 min. Unlike glycerol and glycogen contributions, the contribution from the TCA cycle remained relatively constant throughout the study period in both groups. When examined by two-factor repeated-measures ANOVA, the interaction between glycerol versus glycogen contribution was highly significant for both groups, indicating that the changes in glycogen contribution were primarily dependent on the changes in the contribution of glycerol to glucose production.

## DISCUSSION

We probed multiple biochemical processes in humans with fatty liver using the enrichment and distribution of ^13^C and ^2^H in plasma TG-glycerol and glucose after oral administrations of [U-^13^C_3_]glycerol and ^2^H_2_O. Subclinical hepatic steatosis was associated with enhanced and more rapid metabolism of [U-^13^C_3_]glycerol through pathways that intersect the TCA cycle, as demonstrated by the ^13^C distribution in TG-glycerol. When examining the incorporation of ^13^C and ^2^H into glucose in those with fatty liver, we observed a marked delay in both time to peak ^13^C enrichment and onset of utilization of exogenous glycerol for gluconeogenesis. The present work demonstrates that hepatic steatosis coexists with altered hepatic triglyceride and glucose metabolism and that these metabolic derangements are observed using simple stable isotope probes under non-steady-state conditions.

The fraction of triglycerides in plasma that have [^13^C]glycerol in the backbones (TG-[^13^C]glycerol) is sensitive to the pool size of metabolically active triglycerides in the body. Earlier work indicated that about 40% of total hepatic triglycerides turn over quickly (16, 17). As a simple approximation, we consider that this pool of triglycerides plus plasma triglycerides are metabolically active over a short time scale (minutes to hours) and that the remainder of body triglycerides do not turn over rapidly and therefore do not dilute TG-[^13^C]glycerol significantly in the relatively short duration after an oral load of [U-^13^C_3_]glycerol. The present study demonstrated that the concentration of plasma TG-[^13^C]glycerol was not different between the two groups up to 240 min after [U-^13^C_3_]glycerol administration (Fig. 1F). This implies that triglyceride production rate into the circulation did not differ between the groups. Thus, the lower fraction of plasma TG-[^13^C]glycerol in subjects with steatosis (Fig. 1E) should be the consequence of greater isotopic dilution by metabolically active IHTG and plasma triglycerides in the high-IHTG group.

Glycerol metabolism through the TCA cycle differed between the low- and high-IHTG groups. The fatty liver group had higher fractions of [U-^13^C_3_]glycerol metabolism through the TCA cycle based on TG-[^13^C_2_]glycerol (Fig. 2B; *P* < 0.001) and [5,6-^13^C_2_]glucose (Fig. 4C, D; not significant) production. This demonstrates that alteration of normal mitochondrial functions occurs prior to common clinical manifestations of NAFLD. Unexpectedly we also found that the fraction of indirect contribution through the TCA cycle increased linearly over time as detected by either the ^13^C-labeling pattern in plasma glucose (Fig. 4D) or the ^13^C-labeling pattern in the backbones of plasma triglycerides (Fig. 2B). The steady increase in the indirect contribution indicates that ^13^C-labeled metabolites derived from [U-^13^C_3_]glycerol are entering the TCA cycle for biosynthesis, even after oral glycerol has been absorbed. For example, triple-labeled glucose derived from the tracer can be utilized in muscles through the circulation. Subsequent glycolysis in muscles produces [U-^13^C_3_]lactate, which is an important substrate for hepatic gluconeogenic processes, producing double-labeled glucose or glycerol backbones of triglycerides.

It was not surprising to observe enhanced glycerol contribution to glucose after the load of [U-^13^C_3_]glycerol. However, it was interesting that increased glycerol contribution was accompanied with decreased glycogen contribution, while the contribution from the TCA cycle remained relatively unchanged (Fig. 6). Previously, glycerol supply was reported to decrease the fractional contributions from other gluconeogenic precursors such as amino acids (18, 19). The present study supports the concept of hepatic autoregulation: the supply of a gluconeogenic precursor increases the fractional contribution from the precursor to glucose production, while it suppresses other contributions. Here, we demonstrated that the contribution of glycogen to plasma glucose was decreased in concordance with the transient increase of glycerol contribution after an oral load of glycerol. Another interesting observation was the delayed response in glucose production after the glycerol load among patients with fatty liver. The responses in glycerol and glycogen contributions were much slower in patients with fatty liver, suggesting metabolic inflexibility. These observations, based on ^2^H NMR of glucose, were confirmed by ^13^C NMR of glucose with initial, lower gluconeogenesis from [U-^13^C_3_]glycerol in patients with fatty liver (Fig. 3C, D). High plasma glucose concentration may contribute to low ^13^C enrichment in glucose. However, the ^13^C enrichment at 30 min was much lower in subjects with steatosis (Fig. 3C), which cannot be fully explained only by the very small difference in plasma glucose concentrations (Fig. 3B). A delayed metabolic response in glucose production in the fatty liver group was obvious, but there was no corresponding delay in triglyceride synthesis.

Aside from triglyceride accumulation in the liver, there were few differences in clinical parameters between the low- and high-IHTG groups (Table 1). These similarities are anticipated because subjects were selected for the absence of overt metabolic disorders. As others have shown (2), there is a continuum of fat burden even among asymptomatic individuals. The current observations among subjects with subclinical hepatic steatosis such as increased metabolism through the TCA cycle or metabolic inflexibility are consistent with earlier observations among subjects with typical insulin-resistant NAFLD associated with obesity. Increased [U-^13^C_3_]glycerol metabolism through the TCA cycle is consistent with a common feature of hepatic insulin resistance, such as enhanced gluconeogenesis from the TCA cycle. The terms of “metabolic inflexibility” was introduced to describe an impaired ability to switch from fat to glucose oxidation in skeletal muscles of diabetic patients under insulin-stimulated conditions (20). In the present study, the slower adjustment in glucose production after a load of glycerol could be a part of metabolic inflexibility characterized in patients with NAFLD.

In summary, we demonstrated altered lipogenic and gluconeogenic processes in humans with simple hepatic steatosis after oral loads of [U-^13^C_3_]glycerol and ^2^H_2_O. Although these patients had subclinical NAFLD, excess hepatic triglycerides were associated with altered biochemical processes, including increased utilization of pathways intersecting the TCA cycle and less flexibility in carbohydrate metabolism in response to the glycerol load. Because metabolic processes probed in the present work are highly relevant in many forms of liver diseases, further application of the current method to an advanced liver disease or intervention should be considered.
